# Crystal structures and Hirshfeld surface analyses of bis­[*N*,*N*-bis­(2-meth­oxy­eth­yl)di­thio­carbamato-κ^2^ *S*,*S*′]di-*n*-butyl­tin(IV) and [*N*-(2-meth­oxy­eth­yl)-*N*-methyl­dithio­carbamato-κ^2^ *S*,*S*′]tri­phenyl­tin(IV)

**DOI:** 10.1107/S2056989018001901

**Published:** 2018-02-07

**Authors:** Rapidah Mohamad, Normah Awang, Nurul Farahana Kamaludin, Mukesh M. Jotani, Edward R. T. Tiekink

**Affiliations:** a Biomedical Science Programme, School of Diagnostic and Applied Health Sciences, Faculty of Health Sciences, Universiti Kebangsaan Malaysia, Jalan Raja Muda Abdul Aziz, 50300 Kuala Lumpur, Malaysia; b Environmental Health and Industrial Safety Programme, School of Diagnostic and Applied Health Sciences, Faculty of Health Sciences, Universiti Kebangsaan Malaysia, Jalan Raja Muda Abdul Aziz, 50300 Kuala Lumpur, Malaysia; c Department of Physics, Bhavan’s Sheth R. A. College of Science, Ahmedabad, Gujarat 380 001, India; d Research Centre for Chemical Crystallography, School of Science and Technology, Sunway University, 47500 Bandar Sunway, Selangor Darul Ehsan, Malaysia

**Keywords:** crystal structure, organotin, di­thio­carbamate, Hirshfeld surface analysis

## Abstract

The crystal and mol­ecular structures of the two title organotin di­thio­carbamate compounds, [Sn(C_4_H_9_)_2_(C_7_H_14_NO_2_S_2_)_2_], (I), and [Sn(C_6_H_5_)_3_(C_5_H_10_NOS_2_)], (II), are described. Both structures feature asymmetrically bound di­thio­carbamate ligands leading to a skew-trapezoidal bipyramidal geometry for the metal atom in (I) and a distorted tetra­hedral geometry in (II). The complete mol­ecule of (I) is generated by a crystallographic twofold axis (Sn site symmetry 2). In the crystal of (I), mol­ecules self-assemble into a supra­molecular array parallel to (10-1) *via* methyl­ene-C—H⋯O(meth­oxy) inter­actions. In the crystal of (II), supra­molecular dimers are formed *via* pairs of weak phenyl-C—H⋯π(phen­yl) contacts. In each of (I) and (II), the specified assemblies connect into a three-dimensional architecture without directional inter­actions between them. Hirshfeld surface analyses confirm the importance of H⋯H contacts in the mol­ecular packing of each of (I) and (II), and in the case of (I), highlight the importance of short meth­oxy-H⋯H(but­yl) contacts between layers.

## Abstract 2

The coordination geometry in (*n*-Bu)_2_Sn[S_2_CN(CH_2_CH_2_OCH_3_)_2_]_2_, (I), is based on a skewed trapezoidal bipyramid, while that in (C_6_H_5_)_3_Sn[S_2_CN(CH_3_)CH_2_CH_2_OCH_3_], (II), is based on a tetra­hedron. In the crystal of (I), supra­molecular layers parallel to (10-1) are sustained by methyl­ene-C—H⋯O(meth­oxy) inter­actions, while in (II), centrosymmetric dimers are formed *via* pairwise weak phenyl-C—H⋯π(phen­yl) contacts.

## Chemical context   

While formerly the purview of all-alkyl substituents (Hogarth, 2005 ▸; Heard, 2005 ▸), recent work in the chemistry of di­thio­carbamate ligands, ^−^S_2_CN(*R*)*R*′, has increasingly seen the inclusion of oxygen atoms in these N-bound groups (Hogarth *et al.*, 2009 ▸), leading to different chemistry/biochemistry. Oxygen can be present as a hydroxyl group, giving rise to supra­molecular aggregation patterns based on hydrogen bonding for otherwise non-aggregating species (Tan *et al.*, 2016 ▸; Jotani *et al.*, 2017 ▸) or as an ether, giving rise to compounds with biological activity (Ferreira *et al.*, 2012 ▸). Organotin di­thio­carbamates have long been known to possess biological activity, in particular as anti-tumour and anti-bacterial agents (Tiekink, 2008 ▸). In keeping with the aforementioned, several recent studies have appeared investigating the biological activity of metal di­thio­carbamates where the ligand contains at least one 2-meth­oxy­ethyl substituent (Khan *et al.*, 2013 ▸, 2016 ▸), including anti-bacterial potential of organo­tins (Mohamad, Awang, Kamaludin & Abu Bakar, 2016 ▸; Mohamad, Awang & Kamaludin, 2016 ▸). The latter studies have been augmented by several structural investigations in recent times (Mohamad, Awang, Jotani & Tiekink, 2016 ▸; Mohamad, Awang, Kamaludin, Jotani *et al.*, 2016 ▸; Mohamad *et al.*, 2017 ▸). In a continuation of these structural studies, herein, the crystal and mol­ecular structures of (*n*-Bu)_2_Sn[S_2_CN(CH_2_CH_2_OCH_3_)_2_]_2_ (I) and (C_6_H_5_)_3_Sn[S_2_CN(CH_3_)CH_2_CH_2_OCH_3_] (II) are reported along with a Hirshfeld surface analysis to provide more details on the mol­ecular packing, which generally lacks directional inter­molecular inter­actions.
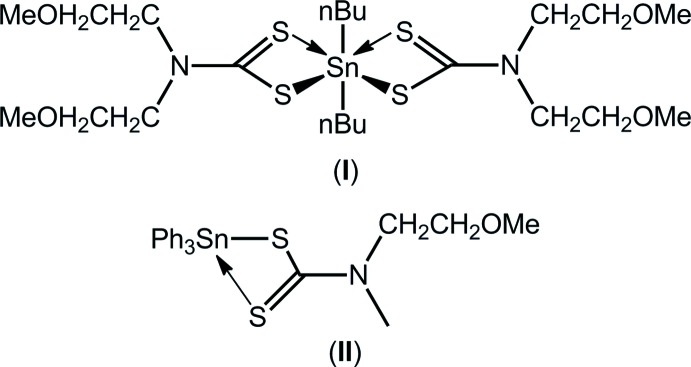


### Structural commentary   

**Figure 1:**
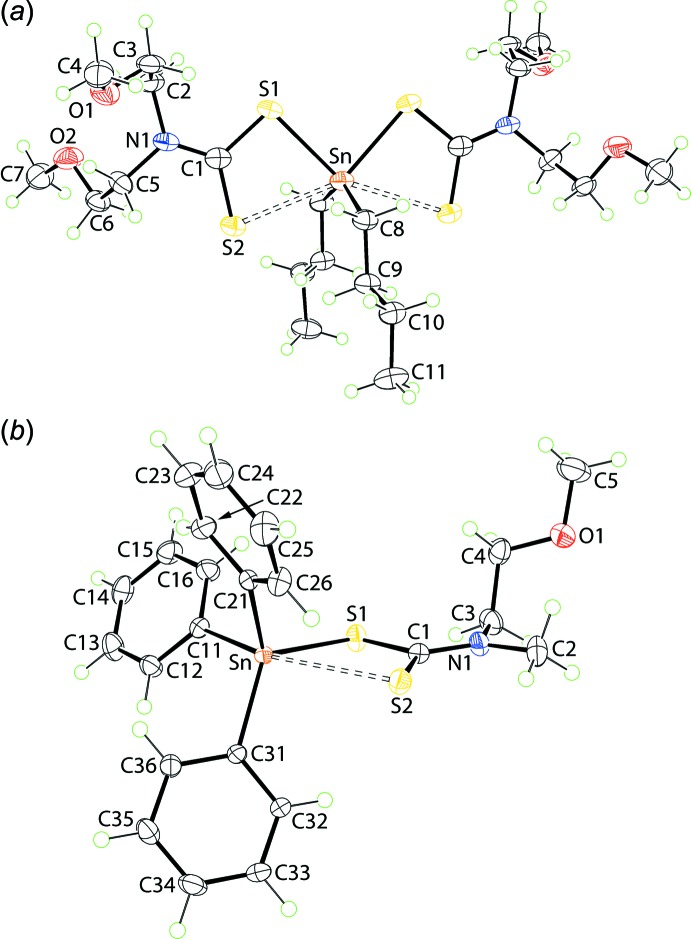
The mol­ecular structures of (*a*) (I) and (*b*) (II), showing the atom-labelling schemes and displacement ellipsoids at the 50% probability level. Unlabelled atoms in (*a*) are related by the symmetry operation *x*, *y*, 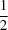 − *z*.

The tin atom in (I), Fig. 1 ▸ *a*, lies on a crystallographic twofold axis so that the asymmetric unit comprises half a mol­ecule. The di­thio­carbamate ligand coordinates to the tin atom with quite disparate Sn—S bond lengths with Δ(Sn—S) = *d*(Sn—S_long_) − (Sn—S)_short_ = 0.38 Å, Table 1 ▸. The disparity in the Sn—S bond lengths is reflected in systematic differences in the C—S bonds lengths with the bond associated with the stronger Sn—S1 bond being significantly longer, *i.e*. by about 0.03 Å, than the C—S bond associated with the weaker Sn—S2 bond. The coordination environment is completed by two α-carbon atoms of the *n*-butyl substituents. The resultant C_2_S_4_ donor set defines a skew-trapezoidal bipyramidal geometry with the tin-bound organic substituents lying over the weaker Sn—S2 bonds, which subtend an angle at the tin atom approximately 50° wider than that subtended by the S1 atoms, Table 1 ▸. The 2-meth­oxy­ethyl groups lie to either side of the S_2_CN residue and have very similar conformations, as seen in the values of the C1—N1—C2—C3, N1—C2—C3—O1 and C2—C3—O1—C4 torsion angles of −94.1 (4), −67.4 (4) and −177.1 (3)°, indicating that − anti-clinal, − syn-clinal and − anti-periplanar descriptors, respectively, are in effect. For the O2-meth­oxy­ethyl group, the equivalent torsion angles are −82.0 (4), −70.3 (4) and −169.1 (3)°. The independent *n*-butyl substituent has an all-*trans* (+ anti-periplanar) conformation, as seen in the values of the Sn—C8—C9—C10 and C8—C9—C10— C11 torsion angles of 172.9 (2) and 176.3 (3)°, respectively.

The mol­ecule in (II), Fig. 1 ▸ *b*, lies on a general position and has a quite distinct coordination geometry owing to the presence of three tin-bound phenyl groups. As for (I), the di­thio­carbamate ligand coordinates in an asymmetric mode with Δ(Sn—S) being 0.55 Å. Consistent with the greater disparity in Sn—S bond lengths, the difference in the associated C—S bond lengths in (II) is greater *cf*. (I), *i.e*. nearly 0.07 Å, Table 2 ▸. The increased asymmetry in the mode of coordination of the di­thio­carbamate ligand in (II), *cf*. (I), is correlated with the reduced Lewis acidity of the tin atom in the triorganotin compound, (II), compared with that in the diorganotin compound, (I). The angles subtended at the tin atom vary from a narrow 64.37 (2)° for the S1—Sn—S2 chelate angle to 155.54 (8)° for S2—Sn—C11. The C_3_S_2_ donor set approximates a trigonal–bipyramidal geometry with the value of τ, an indicator of a five-coordinate coordination geometry, being 0.61, *cf*. 1.0 for an ideal trigonal bipyramid and 0.0 for an ideal square pyramid (Addison *et al.*, 1984 ▸). If the coordination geometry is considered as being based on a C_3_S donor set, the range of tetra­hedral angles is 91.17 (8)°, for S1—Sn—C11, to 119.09 (7)°, for S1—Sn—C31. The C21—Sn—C31 angle, at 115.55 (10)°, is wider by 10° than the other C—Sn—C angles, a result correlated with the close approach of the S2 atom. The 2-meth­oxy­ethyl group has a very similar conformation to the O1-meth­oxy­ethyl group in (I), with the values of the C1—N1—C3—C4, N1—C3—C4—O1 and C5—O1—C4—C3 torsion angles being 95.1 (3), 81.8 (3) and 178.7 (3)°, respectively.

## Supra­molecular features   

**Figure 2:**
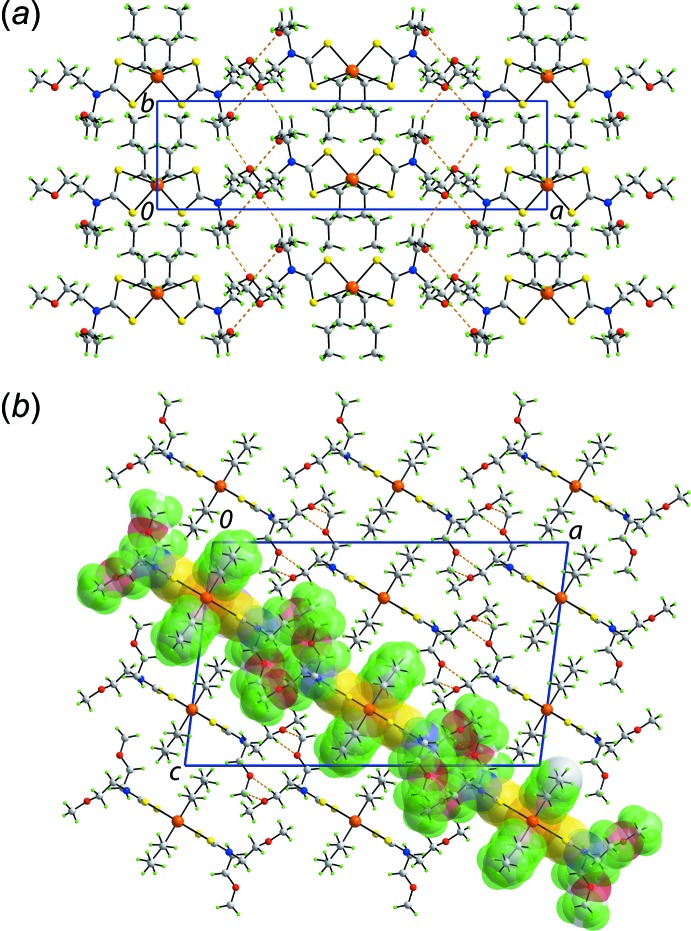
Mol­ecular packing in the crystal of (I): (*a*) supra­molecular layer parallel to (10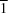) sustained by methyl­ene-C—H⋯O(meth­oxy) inter­actions shown as orange dashed lines and (*b*) a view of the unit-cell contents in projection down the *b* axis, with one layer highlighted in space-filling mode.

Geometric parameters characterizing the inter­molecular inter­actions operating in the crystal structures of (I) and (II) are collected in Tables 3 ▸ and 4 ▸, respectively. The mol­ecular packing of (I) is dominated by methyl­ene-C—H⋯O(meth­oxy) inter­actions whereby each meth­oxy-oxygen atom accepts a single inter­action. Supra­molecular chains form about the twofold axis along *b* so that a supra­molecular array is formed parallel to (10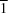), Fig. 2 ▸ *a*. Layers stack with no directional inter­actions between them, Fig. 2 ▸ *b*.

**Figure 3:**
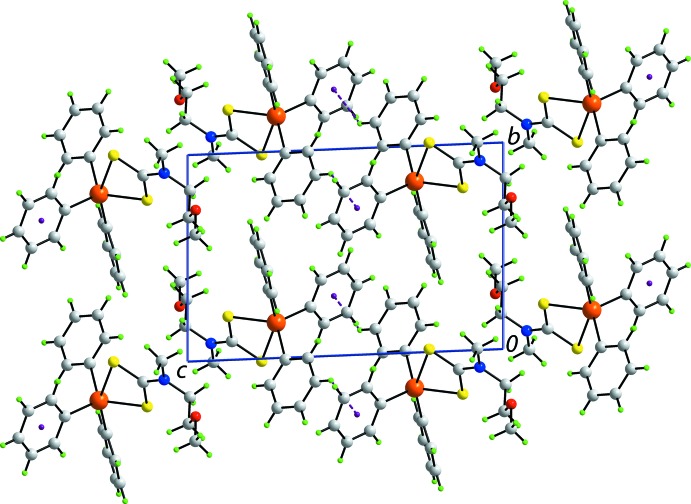
Mol­ecular packing in the crystal of (II): a view of the unit-cell contents in projection down the *a* axis. The phenyl-C—H⋯π(phen­yl) inter­actions are shown as purple dashed lines.

The mol­ecular packing in (II) is largely devoid of directional inter­actions with the only contact rated in *PLATON* (Spek, 2009 ▸) being a phenyl-C—H⋯π(phen­yl) contact. These occur between centrosymmetrically related mol­ecules to form dimeric aggregates which assemble into columns parallel to the *a* axis, Fig. 3 ▸

## Hirshfeld surface analysis   

The Hirshfeld surface calculations for the organotin derivatives (I) and (II) were performed in accord with recent work on related organotin di­thio­carbamate compounds (Mohamad *et al.*, 2017 ▸), and these exhibit different inter­molecular environments as described below.

**Figure 4:**
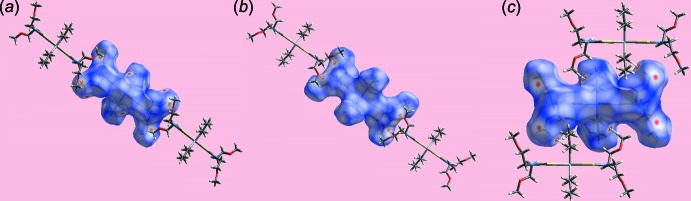
Views of Hirshfeld surface for (I) mapped over *d*_norm_ in the range −0.163 to +1.302, highlighting (*a*) and (*b*) inter­molecular methyl­ene-C—H⋯O(meth­oxy) inter­actions and (*c*) short intra-layer H⋯H contacts between meth­oxy- and butyl-hydrogen atoms H4*B* and H8*A* as sky-blue dashed lines.

**Figure 5:**
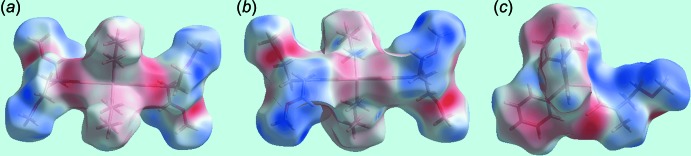
Views of Hirshfeld surface mapped over the electrostatic potential (the red and blue regions represent negative and positive electrostatic potentials, respectively) for: (*a*) and (*b*) a mol­ecule of (I) in the range −0.054 to +0.036 au and (*c*) a mol­ecule of (II) in the range ±0.036 au.

The bright-red spots near each of the meth­oxy-O1 and -O2, and methyl­ene-H4*A* and H6*B* atoms lying on both the sides of twofold symmetry axis on the Hirshfeld surfaces mapped over *d*_norm_ for (I) in Fig. 4 ▸ *a* and *b* represent the dominant inter­molecular C—H⋯O contacts, Table 3 ▸. In addition, the bright-red spots appearing near the meth­oxy-H8*B* and butyl-H8*A* atoms in Fig. 4 ▸ *c* indicate the significant influence of intra-layer H⋯H contacts, Table 5 ▸. On the Hirshfeld surface mapped over the electrostatic potential for (I) shown in Fig. 5 ▸ *a* and *b*, the donors and acceptors are represented with blue and red regions around the respective atoms corresponding to positive and negative potentials, respectively.

**Figure 6:**
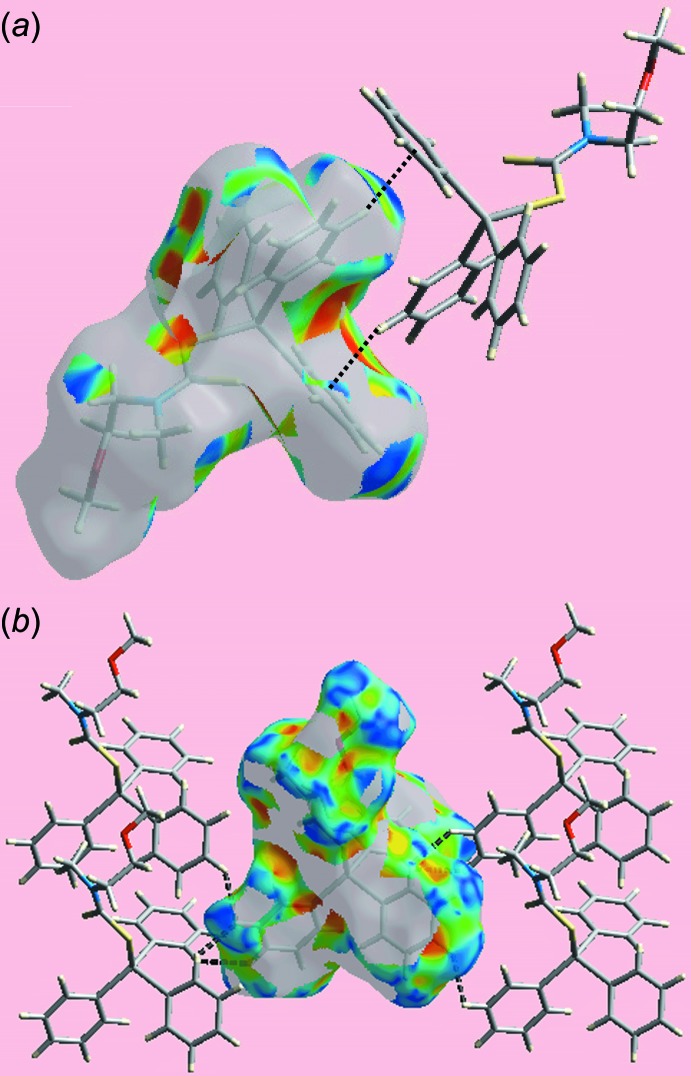
Views of the Hirshfeld surface for (II) mapped with the shape-index property showing (*a*) inter­molecular C—H⋯π/π⋯H—C contacts and (*b*) short inter­atomic H⋯H contacts through black-dashed lines.

The Hirshfeld surfaces mapped over *d*_norm_ for (II) (not shown), indicate the absence of significant directional inter­actions operating in the crystal as no characteristic red spots appear on the surface. The blue and red regions on the Hirshfeld surface mapped over electrostatic potential for (II) in Fig. 5 ▸ *c* are due to polarization of charges near the respective functional groups and do not represent any significant inter­action in the crystal. The weak inter­molecular C—H⋯π contact and intra-layer inter­atomic H⋯H contacts (Table 5 ▸) present in the crystal of (II) are illustrated in Fig. 6 ▸.

**Figure 7:**
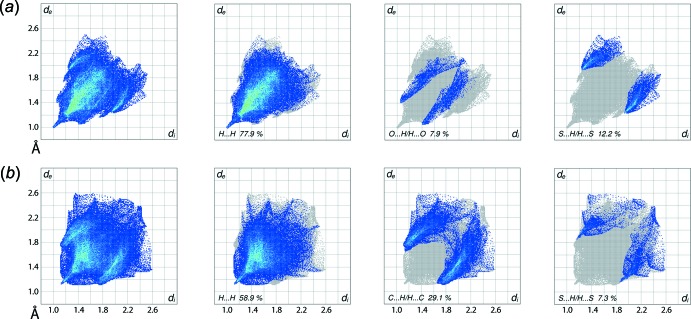
Comparison of the full two-dimensional fingerprint plots for (I) and (II), and the plots delineated into (*a*) H⋯H, O⋯H/H⋯O and S⋯H/H⋯S contacts and (*b*) H⋯H, C⋯H/H⋯C and S⋯H/H⋯S contacts.

The overall two-dimensional fingerprint plots for (I) and (II), Fig. 7 ▸ *a* and *b*, reveal the distinct supra­molecular associations in their crystals. The terminal meth­oxy-ethyl and coordinated *n*-butyl substituents in (I) form significant intra-layer H⋯H contacts in comparison to (II), Table 5 ▸. This fact is also indicated in the fingerprint plots delineated into H⋯H contacts (McKinnon *et al.*, 2007 ▸), showing a short thick spike at *d*_e_ + *d*_i_ ∼ 2.0 Å and the distribution of points with greater density in (*d*_e_, *d*_i_) range ∼1.0 to 1.2 Å for (I) in Fig. 7 ▸ *a*, and a small peak at *d*_e_ + *d*_i_ ∼ 2.2 Å with relatively few points at *d*_e_ + *d*_i_ < 2.4 Å for (II) in Fig. 7 ▸ *b*. The fingerprint plot delineated into O⋯H/H⋯O contacts for (I), Fig. 7 ▸ *a*, characterizes inter­molecular C—H⋯O inter­actions as the pair of forceps-like tips at *d*_e_ + *d*_i_ ∼ 2.5 Å. A low percentage contribution due to O⋯H/H⋯O contacts is noted for (II), as summarized in Table 6 ▸. The relatively high, *i.e.* 29.1%, contribution from C⋯H/H⋯C contacts to the Hirshfeld surfaces of (II) is due to the presence of tin-bound phenyl substituents and the resulting short inter­atomic C⋯H/H⋯C contacts, Table 5 ▸, and inter­molecular C—H⋯π contact, Table 4 ▸, viewed as the pair of peaks at *d*_e_ + *d*_i_ ∼ 2.8 Å and the distribution of points around *d*_e_ + *d*_i_ ∼ 2.9 Å in both the wings of its delineated fingerprint plot, Fig. 7*b* ▸. Although S⋯H/H⋯S contacts have significant contributions to the Hirshfeld surfaces of (I) and (II), as summarized in Table 6 ▸, their inter­atomic distances are farther than sum of their van der Waals radii, *i.e. d*_e_ + *d*_i_ > 3.0 Å, Fig. 7 ▸, and hence do not have a structure-directing influence on the mol­ecular packing. The small contributions from other contacts in (I) and (II) also have negligible impact in the respective crystals.

## Database survey   

It is well documented that organotin di­thio­carbamates, *R*′′_*x*_Sn(S_2_CN*RR*′)_4–*x*_, can adopt a variety of coordination geometries, especially for *x* = 2 (Tiekink, 2008 ▸). The structural motifs for the *x* = 2 series were recently summarized (Zaldi *et al.*, 2017 ▸) and four structural motifs recognized. With a trapezoidal bipyramidal geometry being observed in (I), this structure conforms to the common motif for the *x* = 2 structures. There is one other diorganotin structure with the same di­thio­carbamate ligand, *viz*. the *R*′′ = C_6_H_5_ compound (Mohamad, Awang, Jotani *et al.*, 2016 ▸). This, too, adopts the common trapezoidal bipyramidal geometry although a good number of other derivatives with *R*′′ = Ph adopt octa­hedral geometries, such as in (C_6_H_5_)_2_Sn[S_2_CN(CH_3_)CH_2_CH_2_OCH_3_]_2_ (Muthalib *et al.*, 2014 ▸) featuring the same di­thio­carbamate ligand as in (II). The observed anisobidentate mode of coordination for the di­thio­carbamate ligand in (II) is as expected and in fact is the norm for *x* = 3 structures which may be described as having 4 + 1 coordination geometries (Tiekink, 2008 ▸).

## Synthesis and crystallization   

All chemicals and solvents were used as purchased without purification. The melting points were determined using an automated melting point apparatus (MPA 120 EZ-Melt). Carbon, hydrogen, nitro­gen and sulfur analyses were performed on a Leco CHNS-932 Elemental Analyzer. The IR spectra were obtained on a Perkin Elmer Spectrum GX from 4000 to 400 cm^−1^. NMR spectra were recorded at room temperature on Bruker AVANCE 400 lll HD in CDCl_3_.

**Synthesis of (I):** bis­(2-meth­oxy­eth­yl)amine (Aldrich; 1.48 ml, 10 mmol) dissolved in ethanol (30 ml) was stirred for 30 min. Then, carbon di­sulfide (0.6 ml, 10 mmol) in cold ethanol was added and the resulting mixture was stirred for 2 h. A 25% ammonia solution (1–2 ml) was added to generate basic conditions. Then, di-*n*-butyl­tin(IV) dichloride (Aldrich; 1.52 g, 5 mmol) dissolved in ethanol (20–30 ml) was added dropwise into the solution and stirring was continued for 2 h. All reactions were carried out at 277 K. The precipitate that formed was dried and collected. Colourless prisms were harvested from the slow evaporation of its chloro­form:ethanol (2:1 *v*/*v*) solution. Yield: 72%. M.p. 341–342 K. Elemental analysis: calculated (%): C 40.68, H 7.14, N 4.31, S 19.75. Found (%): C 41.76, H 6.07, N 4.91, S 19.25. IR (KBr cm^−1^): 1487 ν(C—N), 992 ν(C—S), 544 ν(Sn—C), 386 ν(Sn—S). ^1^H NMR (CDCl_3_): δ 4.13 (2H, O—CH_2_); 3.70 (2H, N—CH_2_); 3.35 (3H, O—CH_3_); 1.45–2.05 (6H, Sn—CH_2_—CH_2_—CH_2_–), 0.94 (3H, CH_2_—C*H*_3_). ^13^C NMR (CDCl_3_): δ 201.52 (NCS_2_); 70.07 (O—CH_2_); 55.59 (N—CH_2_); 59.01 (O—CH_3_); 34.26 Sn—CH_2_; 28.55 Sn—CH_2_C*H*_2_; 26.41 Sn—CH_2_CH_2_C*H*_2_; 13.87 CH_2_C*H*_3_. ^119^Sn NMR (CDCl_3_): δ −335.5.

**Synthesis of (II):** The synthesis of (II) was carried out in the same manner as for (I) using (2-meth­oxy­eth­yl)methyl­amine (Santa Cruz Biotechnology; 1.1 ml, 10 mmol) and tri­phenyl­tin(IV) chloride (Merck; 3.85 g, 10 mmol). Crystallization in the form of colourless slabs was from its chloro­form:ethanol (1:2 *v*/*v*) solution. Yield: 78%. M.p. 366-367 K. Elemental analysis: calculated (%): C 53.71, H 4.89, N 2.72, S 12.47. Found (%): C 54.28, H 5.26, N 2.73, S 12.5. IR (KBr cm^−1^): 1477 ν(C—N), 997 ν(C—S), 527 ν(Sn—C), 451 ν(Sn—S). ^1^H NMR (CDCl_3_): δ 7.41–7.82 (15H, Sn—C_6_H_5_); 4.05 (2H, O—CH_2_); 3.71 (2H, N—CH_2_); 3.51 (3H, O—CH_3_); 3.38 (3H, N—CH_3_). ^13^C NMR (CDCl_3_): δ 196.97 (NCS_2_); 128.25–142.28 (C-aromatic); 70.09 (O—CH_2_); 59.06 (N—CH_2_); 58.10 (O—CH_3_); 45.81 (N—CH_3_);. ^119^Sn NMR (CDCl_3_): δ −183.8.

## Refinement   

Crystal data, data collection and structure refinement details are summarized in Table 7 ▸. Carbon-bound H atoms were placed in calculated positions (C—H = 0.95–0.99 Å) and were included in the refinement in the riding-model approximation, with *U*_iso_(H) set to 1.2–1.5*U*_eq_(C). For (I), the maximum and minimum residual electron density peaks of 2.18 and 0.88 e Å^−3^, respectively, were located 0.88 and 1.03 Å from the S1 and Sn atoms, respectively. For (II), the maximum and minimum residual electron density peaks of 2.21 and 1.82 e Å^−3^, respectively, were located 0.96 and 0.76 Å from the Sn atom.

## Supplementary Material

Crystal structure: contains datablock(s) I, II, global. DOI: 10.1107/S2056989018001901/hb7731sup1.cif

Structure factors: contains datablock(s) I. DOI: 10.1107/S2056989018001901/hb7731Isup2.hkl

Structure factors: contains datablock(s) II. DOI: 10.1107/S2056989018001901/hb7731IIsup3.hkl

CCDC references: 1821129, 1821128

Additional supporting information:  crystallographic information; 3D view; checkCIF report

## Figures and Tables

**Table 1:** Selected geometric parameters (Å, °) for (I)

Sn—S1	2.5503 (9)	S1—C1	1.736 (3)
Sn—S2	2.9300 (9)	S2—C1	1.702 (3)
Sn—C8	2.131 (3)		
			
S1—Sn—S2	65.13 (3)	S2—Sn—C8	83.95 (9)
S1—Sn—S1^i^	87.95 (4)	S1—Sn—C8^i^	104.64 (9)
S2—Sn—S2^i^	141.79 (3)	S2—Sn—C8^i^	82.96 (9)
S1—Sn—C8	104.38 (9)	C8—Sn—C8^i^	139.25 (17)

Symmetry code: (i) 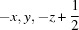.

**Table 2:** Selected geometric parameters (Å, °) for (II)

Sn—S1	2.4711 (7)	Sn—C11	2.162 (3)
Sn—S2	3.0180 (7)	Sn—C21	2.136 (3)
S1—C1	1.755 (3)	Sn—C31	2.133 (2)
S2—C1	1.686 (3)		
			
S1—Sn—S2	64.37 (2)	S2—Sn—C21	87.38 (7)
S1—Sn—C11	91.17 (8)	S2—Sn—C31	87.83 (7)
S1—Sn—C21	115.84 (7)	C11—Sn—C21	104.11 (10)
S1—Sn—C31	119.09 (7)	C11—Sn—C31	105.78 (10)
S2—Sn—C11	155.54 (8)	C21—Sn—C31	115.55 (10)

**Table 3:** Hydrogen-bond geometry (Å, °) for (I) *Cg*1 is the centroid of the N4/C5–C9 ring.

*D*—H⋯*A*	*D*—H	H⋯*A*	*D*⋯*A*	*D*—H⋯*A*
C4—H4*A*⋯O2^ii^	0.98	2.55	3.423 (5)	149
C6—H6*B*⋯O1^iii^	0.99	2.57	3.553 (4)	175

Symmetry codes: (ii) 
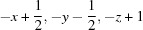
; (iii) 
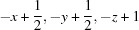
.

**Table 4:** Hydrogen-bond geometry (Å, °) for (II) *Cg*1 is the centroid of the C21–C26 ring.

*D*—H⋯*A*	*D*—H	H⋯*A*	*D*⋯*A*	*D*—H⋯*A*
C35—H35⋯*Cg*1^i^	0.95	2.99	3.760 (3)	139

Symmetry code: (i) 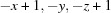.

**Table 5:** Summary of short inter­atomic contacts (Å) in (I)

Contact	Distance	Symmetry operation
(I)		
H4*B*⋯H8*A*	2.00	−*x*, − *y*, 1 − *z*
H5*A*⋯H6*B*	2.21	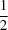 − *x*, 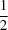 − *y*, 1 − *z*
H8*B*⋯H10*B*	2.37	−*x*, 1 − *y*, 1 − *z*
H10*B*⋯H10*B*	2.37	−*x*, 1 − *y*, 1 − *z*
(II)		
H14⋯H33	2.37	1 + *x*, 1 + *y*, *z*
H16⋯H33	2.25	*x*, 1 + *y*, *z*
H22⋯H34	2.33	*x*, 1 + *y*, *z*
C1⋯H3*B*	2.86	−*x*, − *y*, 2 − *z*
C14⋯H4*A*	2.85	1 − *x*, 1 − *y*, 2 − *z*

**Table 6:** Percentage contributions of inter­atomic contacts to the Hirshfeld surface for (I) and (II)

Contact	% contribution in (I)	% contribution in (II)
H⋯H	77.9	58.9
S⋯H/H⋯S	12.2	7.3
C⋯H/H⋯C	1.6	29.1
O⋯H/H⋯O	7.9	2.5
N⋯H/H⋯N	0.4	0.7
C⋯S/S⋯C	0.0	1.3
S⋯O/O⋯S	0.0	0.1
Sn⋯H/N⋯Sn	0.0	0.1

**Table 7:** Experimental details

	(I)	(II)
Crystal data		
Chemical formula	[Sn(C_4_H_9_)_2_(C_7_H_14_NO_2_S_2_)_2_]	[Sn(C_6_H_5_)_3_(C_5_H_10_NOS_2_)]
*M*_r_	649.54	514.25
Crystal system, space group	Monoclinic, *C*2/*c*	Triclinic, *P* 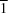
Temperature (K)	173	148
*a*, *b*, *c* (Å)	25.8819 (17), 7.1272 (4), 16.4146 (11)	7.6258 (3), 10.2178 (3), 14.8621 (6)
α, β, γ (°)	90, 97.282 (6), 90	91.976 (3), 90.655 (3), 107.875 (3)
*V* (Å^3^)	3003.5 (3)	1101.19 (7)
*Z*	4	2
Radiation type	Mo *K*α	Mo *K*α
μ (mm^−1^)	1.16	1.36
Crystal size (mm)	0.30 × 0.15 × 0.05	0.50 × 0.30 × 0.30

Data collection		
Diffractometer	Agilent Technologies SuperNova Dual diffractometer with Atlas detector	Agilent Technologies SuperNova Dual diffractometer with Atlas detector
Absorption correction	Multi-scan (*CrysAlis PRO*; Agilent, 2015 ▸)	Multi-scan (*CrysAlis PRO*; Agilent, 2015 ▸)
*T*_min_, *T*_max_	0.247, 1.000	0.479, 1.000
No. of measured, independent and observed [*I* > 2σ(*I*)] reflections	18349, 4631, 3678	11916, 6547, 6089
*R*_int_	0.054	0.046
(sin θ/λ)_max_ (Å^−1^)	0.738	0.739

Refinement		
*R*[*F* ^2^ > 2σ(*F* ^2^)], *wR*(*F* ^2^), *S*	0.048, 0.128, 1.05	0.040, 0.114, 1.09
No. of reflections	4631	6547
No. of parameters	153	255
H-atom treatment	H-atom parameters constrained	H-atom parameters constrained
Δρ_max_, Δρ_min_ (e Å^−3^)	2.18, −0.88	2.21, −1.82

Computer programs: *CrysAlis PRO* (Agilent, 2015 ▸), *SHELXS97* (Sheldrick, 2008 ▸), *SHELXL2014* (Sheldrick, 2015 ▸), *ORTEP-3 for Windows* (Farrugia, 2012 ▸), *DIAMOND* (Brandenburg, 2006 ▸) and *publCIF* (Westrip, 2010 ▸).

## supplementary crystallographic information

### Bis[N,N-bis(2-methoxyethyl)dithiocarbamato-κ2S,S']di-n-butyltin(IV) (I) Crystal data

**Table:**

[Sn(C_4_H_9_)_2_(C_7_H_14_NO_2_S_2_)_2_]	*F*(000) = 1352
*M**_r_* = 649.54	*D*_x_ = 1.436 Mg m^−^^3^
Monoclinic, *C*2/*c*	Mo *K*α radiation, λ = 0.71073 Å
*a* = 25.8819 (17) Å	Cell parameters from 5103 reflections
*b* = 7.1272 (4) Å	θ = 3.9–30.2°
*c* = 16.4146 (11) Å	µ = 1.16 mm^−^^1^
β = 97.282 (6)°	*T* = 173 K
*V* = 3003.5 (3) Å^3^	Prism, colourless
*Z* = 4	0.30 × 0.15 × 0.05 mm

### Bis[N,N-bis(2-methoxyethyl)dithiocarbamato-κ2S,S']di-n-butyltin(IV) (I) Data collection

**Table:**

Agilent Technologies SuperNova Dual diffractometer with Atlas detector	4631 independent reflections
Radiation source: SuperNova (Mo) X-ray Source	3678 reflections with *I* > 2σ(*I*)
Mirror monochromator	*R*_int_ = 0.054
Detector resolution: 10.4041 pixels mm^-1^	θ_max_ = 31.6°, θ_min_ = 3.3°
ω scan	*h* = −36→37
Absorption correction: multi-scan (CrysAlis Pro; Agilent, 2015)	*k* = −9→9
*T*_min_ = 0.247, *T*_max_ = 1.000	*l* = −18→23
18349 measured reflections	

### Bis[N,N-bis(2-methoxyethyl)dithiocarbamato-κ2S,S']di-n-butyltin(IV) (I) Refinement

**Table:**

Refinement on *F*^2^	0 restraints
Least-squares matrix: full	Hydrogen site location: inferred from neighbouring sites
*R*[*F*^2^ > 2σ(*F*^2^)] = 0.048	H-atom parameters constrained
*wR*(*F*^2^) = 0.128	*w* = 1/[σ^2^(*F*_o_^2^) + (0.0581*P*)^2^ + 4.9909*P*] where *P* = (*F*_o_^2^ + 2*F*_c_^2^)/3
*S* = 1.05	(Δ/σ)_max_ < 0.001
4631 reflections	Δρ_max_ = 2.18 e Å^−^^3^
153 parameters	Δρ_min_ = −0.88 e Å^−^^3^

### Bis[N,N-bis(2-methoxyethyl)dithiocarbamato-κ2S,S']di-n-butyltin(IV) (I) Special details

**Table:**

Geometry. All esds (except the esd in the dihedral angle between two l.s. planes) are estimated using the full covariance matrix. The cell esds are taken into account individually in the estimation of esds in distances, angles and torsion angles; correlations between esds in cell parameters are only used when they are defined by crystal symmetry. An approximate (isotropic) treatment of cell esds is used for estimating esds involving l.s. planes.
Refinement. The maximum and minimum residual electron density peaks of 2.18 and 0.88 eÅ^-3^, respectively, were located 0.88 Å and 1.03 Å from the S1 and Sn atoms, respectively.

### Bis[N,N-bis(2-methoxyethyl)dithiocarbamato-κ2S,S']di-n-butyltin(IV) (I) Fractional atomic coordinates and isotropic or equivalent isotropic displacement parameters (Å^2^)

**Table:**

	*x*	*y*	*z*	*U*_iso_*/*U*_eq_	
Sn	0.0000	0.22626 (4)	0.2500	0.03003 (11)	
S1	0.06302 (3)	−0.03124 (11)	0.30644 (5)	0.03538 (19)	
S2	0.09910 (3)	0.36081 (12)	0.33633 (5)	0.03628 (19)	
O1	0.18723 (10)	−0.1322 (4)	0.54586 (15)	0.0420 (6)	
O2	0.26265 (10)	0.1284 (4)	0.34048 (17)	0.0468 (6)	
N1	0.15552 (10)	0.0612 (4)	0.38750 (16)	0.0307 (5)	
C1	0.11053 (12)	0.1264 (4)	0.34726 (18)	0.0300 (6)	
C2	0.16660 (13)	−0.1410 (5)	0.4000 (2)	0.0348 (7)	
H2A	0.2041	−0.1635	0.3970	0.042*	
H2B	0.1465	−0.2127	0.3550	0.042*	
C3	0.15321 (15)	−0.2133 (5)	0.4811 (2)	0.0378 (7)	
H3A	0.1167	−0.1807	0.4872	0.045*	
H3B	0.1566	−0.3516	0.4830	0.045*	
C4	0.17861 (15)	−0.2007 (6)	0.6240 (2)	0.0444 (9)	
H4A	0.1816	−0.3378	0.6247	0.067*	
H4B	0.1437	−0.1645	0.6351	0.067*	
H4C	0.2046	−0.1473	0.6662	0.067*	
C5	0.19574 (13)	0.1927 (5)	0.42487 (19)	0.0329 (7)	
H5A	0.2181	0.1273	0.4695	0.039*	
H5B	0.1785	0.2983	0.4498	0.039*	
C6	0.22976 (13)	0.2705 (5)	0.3646 (2)	0.0342 (7)	
H6A	0.2076	0.3201	0.3157	0.041*	
H6B	0.2511	0.3751	0.3903	0.041*	
C7	0.30157 (16)	0.2029 (6)	0.2963 (3)	0.0475 (9)	
H7A	0.3247	0.2850	0.3323	0.071*	
H7B	0.2851	0.2752	0.2492	0.071*	
H7C	0.3219	0.1000	0.2767	0.071*	
C8	−0.03139 (13)	0.3304 (5)	0.35511 (19)	0.0311 (6)	
H8A	−0.0673	0.2822	0.3543	0.037*	
H8B	−0.0104	0.2810	0.4051	0.037*	
C9	−0.03254 (13)	0.5440 (4)	0.36019 (19)	0.0323 (6)	
H9A	0.0036	0.5920	0.3684	0.039*	
H9B	−0.0498	0.5949	0.3075	0.039*	
C10	−0.06123 (13)	0.6138 (5)	0.42990 (19)	0.0341 (7)	
H10A	−0.0980	0.5726	0.4197	0.041*	
H10B	−0.0454	0.5562	0.4821	0.041*	
C11	−0.05949 (17)	0.8260 (5)	0.4386 (2)	0.0454 (9)	
H11A	−0.0752	0.8838	0.3872	0.068*	
H11B	−0.0232	0.8671	0.4510	0.068*	
H11C	−0.0790	0.8639	0.4834	0.068*	

### Bis[N,N-bis(2-methoxyethyl)dithiocarbamato-κ2S,S']di-n-butyltin(IV) (I) Atomic displacement parameters (Å^2^)

**Table:**

	*U*^11^	*U*^22^	*U*^33^	*U*^12^	*U*^13^	*U*^23^
Sn	0.03662 (18)	0.02227 (17)	0.03257 (17)	0.000	0.00965 (12)	0.000
S1	0.0400 (4)	0.0212 (4)	0.0445 (4)	−0.0060 (3)	0.0036 (3)	−0.0035 (3)
S2	0.0399 (4)	0.0252 (4)	0.0433 (4)	−0.0045 (3)	0.0037 (3)	0.0006 (3)
O1	0.0456 (14)	0.0362 (15)	0.0443 (13)	−0.0097 (11)	0.0063 (11)	0.0091 (11)
O2	0.0485 (15)	0.0295 (14)	0.0657 (16)	−0.0004 (11)	0.0197 (12)	0.0066 (12)
N1	0.0393 (14)	0.0201 (13)	0.0338 (13)	−0.0038 (10)	0.0094 (11)	−0.0006 (10)
C1	0.0391 (16)	0.0268 (16)	0.0267 (13)	−0.0013 (12)	0.0148 (12)	0.0008 (11)
C2	0.0406 (17)	0.0218 (16)	0.0433 (17)	0.0027 (12)	0.0102 (14)	−0.0027 (13)
C3	0.0461 (19)	0.0186 (16)	0.0496 (19)	−0.0032 (12)	0.0101 (15)	0.0015 (13)
C4	0.044 (2)	0.037 (2)	0.052 (2)	−0.0035 (15)	0.0053 (16)	0.0186 (16)
C5	0.0356 (16)	0.0309 (18)	0.0314 (15)	−0.0100 (12)	0.0011 (12)	0.0012 (12)
C6	0.0376 (17)	0.0263 (17)	0.0384 (17)	−0.0085 (12)	0.0038 (13)	0.0054 (12)
C7	0.044 (2)	0.043 (2)	0.058 (2)	−0.0004 (16)	0.0157 (17)	0.0078 (18)
C8	0.0376 (16)	0.0239 (15)	0.0337 (15)	−0.0023 (12)	0.0117 (12)	0.0002 (12)
C9	0.0403 (17)	0.0241 (16)	0.0338 (15)	−0.0014 (12)	0.0093 (13)	−0.0002 (12)
C10	0.0401 (17)	0.0281 (17)	0.0355 (15)	0.0019 (13)	0.0104 (13)	−0.0005 (12)
C11	0.067 (2)	0.0281 (19)	0.0429 (19)	0.0084 (16)	0.0138 (17)	−0.0051 (15)

### Bis[N,N-bis(2-methoxyethyl)dithiocarbamato-κ2S,S']di-n-butyltin(IV) (I) Geometric parameters (Å, º)

**Table:**

Sn—S1	2.5503 (9)	C4—H4C	0.9800
Sn—S1^i^	2.5504 (9)	C5—C6	1.511 (4)
Sn—S2	2.9300 (9)	C5—H5A	0.9900
Sn—S2^i^	2.9300 (9)	C5—H5B	0.9900
Sn—C8	2.131 (3)	C6—H6A	0.9900
Sn—C8^i^	2.131 (3)	C6—H6B	0.9900
S1—C1	1.736 (3)	C7—H7A	0.9800
S2—C1	1.702 (3)	C7—H7B	0.9800
O1—C3	1.415 (4)	C7—H7C	0.9800
O1—C4	1.416 (4)	C8—C9	1.526 (4)
O2—C6	1.412 (4)	C8—H8A	0.9900
O2—C7	1.417 (4)	C8—H8B	0.9900
N1—C1	1.347 (4)	C9—C10	1.524 (4)
N1—C5	1.475 (4)	C9—H9A	0.9900
N1—C2	1.479 (4)	C9—H9B	0.9900
C2—C3	1.508 (5)	C10—C11	1.519 (5)
C2—H2A	0.9900	C10—H10A	0.9900
C2—H2B	0.9900	C10—H10B	0.9900
C3—H3A	0.9900	C11—H11A	0.9800
C3—H3B	0.9900	C11—H11B	0.9800
C4—H4A	0.9800	C11—H11C	0.9800
C4—H4B	0.9800		
			
S1—Sn—S2	65.13 (3)	C6—C5—H5A	108.9
S1—Sn—S1^i^	87.95 (4)	N1—C5—H5B	108.9
S2—Sn—S2^i^	141.79 (3)	C6—C5—H5B	108.9
S1—Sn—C8	104.38 (9)	H5A—C5—H5B	107.7
S2—Sn—C8	83.95 (9)	O2—C6—C5	110.0 (3)
S1—Sn—C8^i^	104.64 (9)	O2—C6—H6A	109.7
C8—Sn—S1^i^	104.64 (9)	C5—C6—H6A	109.7
C8^i^—Sn—S1^i^	104.38 (9)	O2—C6—H6B	109.7
S2—Sn—C8^i^	82.96 (9)	C5—C6—H6B	109.7
S1—Sn—S2^i^	153.08 (3)	H6A—C6—H6B	108.2
C8—Sn—C8^i^	139.25 (17)	O2—C7—H7A	109.5
C1—S1—Sn	93.64 (11)	O2—C7—H7B	109.5
C1—S2—Sn	81.93 (11)	H7A—C7—H7B	109.5
C3—O1—C4	112.6 (3)	O2—C7—H7C	109.5
C6—O2—C7	111.6 (3)	H7A—C7—H7C	109.5
C1—N1—C5	120.4 (3)	H7B—C7—H7C	109.5
C1—N1—C2	123.0 (3)	C9—C8—Sn	113.8 (2)
C5—N1—C2	116.6 (3)	C9—C8—H8A	108.8
N1—C1—S2	121.2 (2)	Sn—C8—H8A	108.8
N1—C1—S1	119.5 (2)	C9—C8—H8B	108.8
S2—C1—S1	119.31 (19)	Sn—C8—H8B	108.8
N1—C2—C3	113.2 (3)	H8A—C8—H8B	107.7
N1—C2—H2A	108.9	C10—C9—C8	112.4 (3)
C3—C2—H2A	108.9	C10—C9—H9A	109.1
N1—C2—H2B	108.9	C8—C9—H9A	109.1
C3—C2—H2B	108.9	C10—C9—H9B	109.1
H2A—C2—H2B	107.8	C8—C9—H9B	109.1
O1—C3—C2	109.4 (3)	H9A—C9—H9B	107.9
O1—C3—H3A	109.8	C11—C10—C9	112.6 (3)
C2—C3—H3A	109.8	C11—C10—H10A	109.1
O1—C3—H3B	109.8	C9—C10—H10A	109.1
C2—C3—H3B	109.8	C11—C10—H10B	109.1
H3A—C3—H3B	108.2	C9—C10—H10B	109.1
O1—C4—H4A	109.5	H10A—C10—H10B	107.8
O1—C4—H4B	109.5	C10—C11—H11A	109.5
H4A—C4—H4B	109.5	C10—C11—H11B	109.5
O1—C4—H4C	109.5	H11A—C11—H11B	109.5
H4A—C4—H4C	109.5	C10—C11—H11C	109.5
H4B—C4—H4C	109.5	H11A—C11—H11C	109.5
N1—C5—C6	113.6 (3)	H11B—C11—H11C	109.5
N1—C5—H5A	108.9		
			
C5—N1—C1—S2	1.4 (4)	C5—N1—C2—C3	83.2 (3)
C2—N1—C1—S2	178.6 (2)	C4—O1—C3—C2	−177.1 (3)
C5—N1—C1—S1	−178.1 (2)	N1—C2—C3—O1	−67.4 (4)
C2—N1—C1—S1	−0.8 (4)	C1—N1—C5—C6	−82.0 (4)
Sn—S2—C1—N1	−179.6 (2)	C2—N1—C5—C6	100.6 (3)
Sn—S2—C1—S1	−0.11 (15)	C7—O2—C6—C5	−169.1 (3)
Sn—S1—C1—N1	179.6 (2)	N1—C5—C6—O2	−70.3 (4)
Sn—S1—C1—S2	0.12 (17)	Sn—C8—C9—C10	172.9 (2)
C1—N1—C2—C3	−94.1 (4)	C8—C9—C10—C11	176.3 (3)

Symmetry code: (i) −*x*, *y*, −*z*+1/2.

### Bis[N,N-bis(2-methoxyethyl)dithiocarbamato-κ2S,S']di-n-butyltin(IV) (I) Hydrogen-bond geometry (Å, º)

Cg1 is the centroid of the N4/C5–C9 ring.

**Table:**

*D*—H···*A*	*D*—H	H···*A*	*D*···*A*	*D*—H···*A*
C4—H4*A*···O2^ii^	0.98	2.55	3.423 (5)	149
C6—H6*B*···O1^iii^	0.99	2.57	3.553 (4)	175

Symmetry codes: (ii) −*x*+1/2, −*y*−1/2, −*z*+1; (iii) −*x*+1/2, −*y*+1/2, −*z*+1.

### [N-(2-Methoxyethyl)-N-methyldithiocarbamato-κ2S,S']triphenyltin(IV) (II) Crystal data

**Table:**

[Sn(C_6_H_5_)_3_(C_5_H_10_NOS_2_)]	*Z* = 2
*M**_r_* = 514.25	*F*(000) = 520
Triclinic, *P*1	*D*_x_ = 1.551 Mg m^−^^3^
*a* = 7.6258 (3) Å	Mo *K*α radiation, λ = 0.71073 Å
*b* = 10.2178 (3) Å	Cell parameters from 8110 reflections
*c* = 14.8621 (6) Å	θ = 4.1–31.4°
α = 91.976 (3)°	µ = 1.36 mm^−^^1^
β = 90.655 (3)°	*T* = 148 K
γ = 107.875 (3)°	Slab, colourless
*V* = 1101.19 (7) Å^3^	0.50 × 0.30 × 0.30 mm

### [N-(2-Methoxyethyl)-N-methyldithiocarbamato-κ2S,S']triphenyltin(IV) (II) Data collection

**Table:**

Agilent Technologies SuperNova Dual diffractometer with Atlas detector	6547 independent reflections
Radiation source: SuperNova (Mo) X-ray Source	6089 reflections with *I* > 2σ(*I*)
Mirror monochromator	*R*_int_ = 0.046
Detector resolution: 10.4041 pixels mm^-1^	θ_max_ = 31.7°, θ_min_ = 3.5°
ω scan	*h* = −11→11
Absorption correction: multi-scan (CrysAlis Pro; Agilent, 2015)	*k* = −15→14
*T*_min_ = 0.479, *T*_max_ = 1.000	*l* = −20→20
11916 measured reflections	

### [N-(2-Methoxyethyl)-N-methyldithiocarbamato-κ2S,S']triphenyltin(IV) (II) Refinement

**Table:**

Refinement on *F*^2^	0 restraints
Least-squares matrix: full	Hydrogen site location: inferred from neighbouring sites
*R*[*F*^2^ > 2σ(*F*^2^)] = 0.040	H-atom parameters constrained
*wR*(*F*^2^) = 0.114	*w* = 1/[σ^2^(*F*_o_^2^) + (0.0671*P*)^2^ + 0.2783*P*] where *P* = (*F*_o_^2^ + 2*F*_c_^2^)/3
*S* = 1.09	(Δ/σ)_max_ = 0.001
6547 reflections	Δρ_max_ = 2.21 e Å^−^^3^
255 parameters	Δρ_min_ = −1.82 e Å^−^^3^

### [N-(2-Methoxyethyl)-N-methyldithiocarbamato-κ2S,S']triphenyltin(IV) (II) Special details

**Table:**

Geometry. All esds (except the esd in the dihedral angle between two l.s. planes) are estimated using the full covariance matrix. The cell esds are taken into account individually in the estimation of esds in distances, angles and torsion angles; correlations between esds in cell parameters are only used when they are defined by crystal symmetry. An approximate (isotropic) treatment of cell esds is used for estimating esds involving l.s. planes.
Refinement. The maximum and minimum residual electron density peaks of 2.21 and 1.82 eÅ^-3^, respectively, were located 0.96 Å and 0.76 Å from the Sn atom.

### [N-(2-Methoxyethyl)-N-methyldithiocarbamato-κ2S,S']triphenyltin(IV) (II) Fractional atomic coordinates and isotropic or equivalent isotropic displacement parameters (Å^2^)

**Table:**

	*x*	*y*	*z*	*U*_iso_*/*U*_eq_	
Sn	0.33155 (2)	0.17167 (2)	0.71926 (2)	0.01646 (7)	
S1	0.22626 (10)	0.21030 (8)	0.87168 (5)	0.02514 (15)	
S2	−0.04590 (10)	−0.01260 (7)	0.76544 (5)	0.02473 (15)	
O1	−0.3089 (3)	0.2686 (2)	1.02520 (19)	0.0379 (6)	
N1	−0.1129 (3)	0.0832 (3)	0.92564 (17)	0.0254 (5)	
C1	0.0058 (4)	0.0900 (3)	0.85949 (19)	0.0211 (5)	
C2	−0.3028 (4)	−0.0073 (4)	0.9179 (3)	0.0358 (7)	
H2A	−0.3842	0.0464	0.9015	0.054*	
H2B	−0.3399	−0.0496	0.9757	0.054*	
H2C	−0.3118	−0.0794	0.8713	0.054*	
C3	−0.0635 (4)	0.1730 (4)	1.0074 (2)	0.0302 (6)	
H3A	0.0717	0.2002	1.0174	0.036*	
H3B	−0.1218	0.1203	1.0596	0.036*	
C4	−0.1216 (4)	0.3017 (3)	1.0033 (2)	0.0293 (6)	
H4A	−0.0459	0.3734	1.0464	0.035*	
H4B	−0.1032	0.3380	0.9421	0.035*	
C5	−0.3705 (6)	0.3865 (4)	1.0244 (3)	0.0456 (9)	
H5A	−0.2985	0.4559	1.0689	0.068*	
H5B	−0.5011	0.3603	1.0395	0.068*	
H5C	−0.3542	0.4246	0.9644	0.068*	
C11	0.5881 (4)	0.3348 (3)	0.74340 (19)	0.0210 (5)	
C12	0.7602 (4)	0.3153 (3)	0.7336 (2)	0.0249 (6)	
H12	0.7669	0.2264	0.7169	0.030*	
C13	0.9229 (4)	0.4240 (4)	0.7479 (2)	0.0321 (7)	
H13	1.0389	0.4091	0.7404	0.039*	
C14	0.9145 (4)	0.5536 (3)	0.7729 (2)	0.0308 (6)	
H14	1.0250	0.6279	0.7821	0.037*	
C15	0.7455 (4)	0.5753 (3)	0.7846 (2)	0.0299 (6)	
H15	0.7397	0.6641	0.8021	0.036*	
C16	0.5846 (4)	0.4662 (3)	0.7704 (2)	0.0262 (6)	
H16	0.4691	0.4814	0.7793	0.031*	
C21	0.2016 (4)	0.2414 (3)	0.61084 (18)	0.0188 (5)	
C22	0.2874 (4)	0.3708 (3)	0.5777 (2)	0.0262 (6)	
H22	0.3998	0.4270	0.6050	0.031*	
C23	0.2114 (5)	0.4192 (4)	0.5050 (2)	0.0345 (7)	
H23	0.2704	0.5083	0.4838	0.041*	
C24	0.0504 (5)	0.3373 (4)	0.4643 (2)	0.0370 (7)	
H24	−0.0018	0.3700	0.4148	0.044*	
C25	−0.0359 (5)	0.2075 (4)	0.4951 (2)	0.0344 (7)	
H25	−0.1463	0.1509	0.4663	0.041*	
C26	0.0387 (4)	0.1600 (3)	0.5682 (2)	0.0263 (6)	
H26	−0.0219	0.0712	0.5894	0.032*	
C31	0.4062 (3)	−0.0098 (3)	0.69017 (18)	0.0175 (5)	
C32	0.3456 (4)	−0.1288 (3)	0.7390 (2)	0.0272 (6)	
H32	0.2659	−0.1323	0.7881	0.033*	
C33	0.4018 (5)	−0.2424 (3)	0.7156 (3)	0.0360 (8)	
H33	0.3608	−0.3231	0.7493	0.043*	
C34	0.5168 (4)	−0.2389 (3)	0.6439 (3)	0.0312 (7)	
H34	0.5535	−0.3173	0.6281	0.037*	
C35	0.5782 (4)	−0.1216 (3)	0.5952 (2)	0.0255 (6)	
H35	0.6573	−0.1190	0.5460	0.031*	
C36	0.5237 (4)	−0.0067 (3)	0.61873 (19)	0.0212 (5)	
H36	0.5673	0.0745	0.5857	0.025*	

### [N-(2-Methoxyethyl)-N-methyldithiocarbamato-κ2S,S']triphenyltin(IV) (II) Atomic displacement parameters (Å^2^)

**Table:**

	*U*^11^	*U*^22^	*U*^33^	*U*^12^	*U*^13^	*U*^23^
Sn	0.01712 (11)	0.01286 (10)	0.01959 (11)	0.00482 (7)	0.00283 (7)	0.00054 (7)
S1	0.0219 (3)	0.0277 (3)	0.0224 (3)	0.0028 (3)	0.0036 (2)	−0.0031 (3)
S2	0.0239 (3)	0.0231 (3)	0.0250 (3)	0.0041 (3)	0.0038 (3)	0.0000 (3)
O1	0.0331 (12)	0.0285 (11)	0.0553 (16)	0.0134 (10)	0.0124 (11)	0.0057 (11)
N1	0.0239 (12)	0.0295 (12)	0.0249 (12)	0.0108 (10)	0.0072 (9)	0.0043 (10)
C1	0.0216 (12)	0.0201 (12)	0.0233 (13)	0.0085 (10)	0.0035 (10)	0.0037 (10)
C2	0.0258 (15)	0.0372 (17)	0.0429 (19)	0.0065 (13)	0.0144 (13)	0.0060 (14)
C3	0.0348 (16)	0.0395 (17)	0.0213 (13)	0.0183 (14)	0.0079 (11)	0.0027 (12)
C4	0.0316 (15)	0.0277 (14)	0.0259 (14)	0.0051 (12)	0.0076 (12)	0.0003 (11)
C5	0.047 (2)	0.0374 (19)	0.060 (3)	0.0249 (17)	−0.0004 (18)	−0.0011 (18)
C11	0.0195 (12)	0.0174 (11)	0.0248 (13)	0.0038 (10)	0.0036 (10)	0.0029 (9)
C12	0.0225 (13)	0.0212 (13)	0.0316 (15)	0.0079 (11)	0.0026 (11)	−0.0024 (11)
C13	0.0185 (13)	0.0373 (17)	0.0381 (17)	0.0052 (12)	0.0043 (12)	−0.0009 (13)
C14	0.0248 (14)	0.0289 (15)	0.0293 (15)	−0.0053 (12)	0.0018 (12)	−0.0011 (12)
C15	0.0336 (16)	0.0176 (12)	0.0348 (16)	0.0025 (11)	0.0007 (12)	−0.0020 (11)
C16	0.0243 (14)	0.0192 (13)	0.0344 (16)	0.0059 (11)	0.0022 (11)	−0.0018 (11)
C21	0.0201 (12)	0.0178 (11)	0.0201 (12)	0.0077 (10)	0.0060 (9)	0.0021 (9)
C22	0.0267 (14)	0.0212 (13)	0.0312 (15)	0.0075 (11)	0.0026 (11)	0.0054 (11)
C23	0.0418 (18)	0.0308 (16)	0.0348 (17)	0.0155 (14)	0.0069 (14)	0.0137 (13)
C24	0.0381 (18)	0.050 (2)	0.0303 (16)	0.0225 (16)	0.0043 (13)	0.0139 (15)
C25	0.0301 (16)	0.0466 (19)	0.0252 (15)	0.0096 (14)	−0.0025 (12)	0.0034 (13)
C26	0.0234 (13)	0.0285 (14)	0.0242 (14)	0.0035 (11)	0.0018 (11)	0.0027 (11)
C31	0.0161 (11)	0.0140 (10)	0.0219 (12)	0.0039 (9)	0.0019 (9)	0.0016 (9)
C32	0.0269 (14)	0.0191 (13)	0.0389 (16)	0.0103 (11)	0.0141 (12)	0.0109 (11)
C33	0.0344 (17)	0.0191 (13)	0.059 (2)	0.0131 (12)	0.0178 (15)	0.0148 (14)
C34	0.0271 (14)	0.0196 (13)	0.0492 (19)	0.0111 (11)	0.0036 (13)	−0.0033 (13)
C35	0.0233 (13)	0.0269 (14)	0.0271 (14)	0.0094 (11)	0.0042 (11)	−0.0049 (11)
C36	0.0212 (12)	0.0199 (12)	0.0220 (12)	0.0054 (10)	0.0036 (10)	0.0006 (10)

### [N-(2-Methoxyethyl)-N-methyldithiocarbamato-κ2S,S']triphenyltin(IV) (II) Geometric parameters (Å, º)

**Table:**

Sn—S1	2.4711 (7)	C13—H13	0.9500
Sn—S2	3.0180 (7)	C14—C15	1.385 (5)
S1—C1	1.755 (3)	C14—H14	0.9500
S2—C1	1.686 (3)	C15—C16	1.390 (4)
Sn—C11	2.162 (3)	C15—H15	0.9500
Sn—C21	2.136 (3)	C16—H16	0.9500
Sn—C31	2.133 (2)	C21—C22	1.393 (4)
O1—C4	1.408 (4)	C21—C26	1.396 (4)
O1—C5	1.420 (4)	C22—C23	1.395 (4)
N1—C1	1.333 (4)	C22—H22	0.9500
N1—C2	1.460 (4)	C23—C24	1.375 (5)
N1—C3	1.470 (4)	C23—H23	0.9500
C2—H2A	0.9800	C24—C25	1.384 (5)
C2—H2B	0.9800	C24—H24	0.9500
C2—H2C	0.9800	C25—C26	1.389 (4)
C3—C4	1.514 (4)	C25—H25	0.9500
C3—H3A	0.9900	C26—H26	0.9500
C3—H3B	0.9900	C31—C36	1.392 (4)
C4—H4A	0.9900	C31—C32	1.393 (4)
C4—H4B	0.9900	C32—C33	1.389 (4)
C5—H5A	0.9800	C32—H32	0.9500
C5—H5B	0.9800	C33—C34	1.383 (5)
C5—H5C	0.9800	C33—H33	0.9500
C11—C12	1.395 (4)	C34—C35	1.379 (5)
C11—C16	1.396 (4)	C34—H34	0.9500
C12—C13	1.396 (4)	C35—C36	1.395 (4)
C12—H12	0.9500	C35—H35	0.9500
C13—C14	1.383 (5)	C36—H36	0.9500
			
S1—Sn—S2	64.37 (2)	C14—C13—C12	119.8 (3)
S1—Sn—C11	91.17 (8)	C14—C13—H13	120.1
S1—Sn—C21	115.84 (7)	C12—C13—H13	120.1
S1—Sn—C31	119.09 (7)	C13—C14—C15	120.2 (3)
S2—Sn—C11	155.54 (8)	C13—C14—H14	119.9
S2—Sn—C21	87.38 (7)	C15—C14—H14	119.9
S2—Sn—C31	87.83 (7)	C14—C15—C16	119.5 (3)
C11—Sn—C21	104.11 (10)	C14—C15—H15	120.2
C11—Sn—C31	105.78 (10)	C16—C15—H15	120.2
C21—Sn—C31	115.55 (10)	C15—C16—C11	121.8 (3)
C1—S1—Sn	96.57 (10)	C15—C16—H16	119.1
C1—S2—Sn	79.96 (10)	C11—C16—H16	119.1
C4—O1—C5	111.3 (3)	C22—C21—C26	118.1 (3)
C1—N1—C2	121.6 (3)	C22—C21—Sn	118.9 (2)
C1—N1—C3	121.8 (3)	C26—C21—Sn	122.9 (2)
C2—N1—C3	116.4 (2)	C21—C22—C23	121.1 (3)
N1—C1—S2	122.9 (2)	C21—C22—H22	119.4
N1—C1—S1	118.3 (2)	C23—C22—H22	119.4
S2—C1—S1	118.72 (16)	C24—C23—C22	119.7 (3)
N1—C2—H2A	109.5	C24—C23—H23	120.2
N1—C2—H2B	109.5	C22—C23—H23	120.2
H2A—C2—H2B	109.5	C23—C24—C25	120.3 (3)
N1—C2—H2C	109.5	C23—C24—H24	119.9
H2A—C2—H2C	109.5	C25—C24—H24	119.9
H2B—C2—H2C	109.5	C24—C25—C26	120.1 (3)
N1—C3—C4	113.6 (3)	C24—C25—H25	120.0
N1—C3—H3A	108.8	C26—C25—H25	120.0
C4—C3—H3A	108.8	C25—C26—C21	120.7 (3)
N1—C3—H3B	108.8	C25—C26—H26	119.6
C4—C3—H3B	108.8	C21—C26—H26	119.6
H3A—C3—H3B	107.7	C36—C31—C32	119.1 (2)
O1—C4—C3	108.7 (3)	C36—C31—Sn	117.20 (19)
O1—C4—H4A	109.9	C32—C31—Sn	123.70 (19)
C3—C4—H4A	109.9	C33—C32—C31	119.9 (3)
O1—C4—H4B	109.9	C33—C32—H32	120.0
C3—C4—H4B	109.9	C31—C32—H32	120.0
H4A—C4—H4B	108.3	C34—C33—C32	120.6 (3)
O1—C5—H5A	109.5	C34—C33—H33	119.7
O1—C5—H5B	109.5	C32—C33—H33	119.7
H5A—C5—H5B	109.5	C35—C34—C33	120.0 (3)
O1—C5—H5C	109.5	C35—C34—H34	120.0
H5A—C5—H5C	109.5	C33—C34—H34	120.0
H5B—C5—H5C	109.5	C34—C35—C36	119.7 (3)
C12—C11—C16	117.5 (3)	C34—C35—H35	120.1
C12—C11—Sn	123.0 (2)	C36—C35—H35	120.1
C16—C11—Sn	119.5 (2)	C31—C36—C35	120.6 (3)
C11—C12—C13	121.2 (3)	C31—C36—H36	119.7
C11—C12—H12	119.4	C35—C36—H36	119.7
C13—C12—H12	119.4		
			
C2—N1—C1—S2	−5.0 (4)	C12—C11—C16—C15	1.9 (5)
C3—N1—C1—S2	179.3 (2)	Sn—C11—C16—C15	−178.3 (2)
C2—N1—C1—S1	175.7 (2)	C26—C21—C22—C23	1.3 (4)
C3—N1—C1—S1	0.0 (4)	Sn—C21—C22—C23	177.9 (2)
Sn—S2—C1—N1	175.2 (2)	C21—C22—C23—C24	−1.1 (5)
Sn—S2—C1—S1	−5.46 (14)	C22—C23—C24—C25	0.0 (5)
Sn—S1—C1—N1	−174.0 (2)	C23—C24—C25—C26	0.8 (5)
Sn—S1—C1—S2	6.62 (16)	C24—C25—C26—C21	−0.6 (5)
C1—N1—C3—C4	95.1 (3)	C22—C21—C26—C25	−0.4 (4)
C2—N1—C3—C4	−80.8 (3)	Sn—C21—C26—C25	−176.9 (2)
C5—O1—C4—C3	178.7 (3)	C36—C31—C32—C33	−0.4 (5)
N1—C3—C4—O1	81.8 (3)	Sn—C31—C32—C33	−179.7 (3)
C16—C11—C12—C13	−1.7 (5)	C31—C32—C33—C34	−0.4 (5)
Sn—C11—C12—C13	178.5 (2)	C32—C33—C34—C35	0.6 (6)
C11—C12—C13—C14	0.5 (5)	C33—C34—C35—C36	0.0 (5)
C12—C13—C14—C15	0.6 (5)	C32—C31—C36—C35	0.9 (4)
C13—C14—C15—C16	−0.4 (5)	Sn—C31—C36—C35	−179.7 (2)
C14—C15—C16—C11	−0.9 (5)	C34—C35—C36—C31	−0.8 (4)

### [N-(2-Methoxyethyl)-N-methyldithiocarbamato-κ2S,S']triphenyltin(IV) (II) Hydrogen-bond geometry (Å, º)

Cg1 is the centroid of the C21–C26 ring.

**Table:**

*D*—H···*A*	*D*—H	H···*A*	*D*···*A*	*D*—H···*A*
C35—H35···*Cg*1^i^	0.95	2.99	3.760 (3)	139

Symmetry code: (i) −*x*+1, −*y*, −*z*+1.
